# The Dentists' Future

**Published:** 1877-02

**Authors:** 


### EDITORIAL, ETC.

---

*The Dentists' Future.*—Under this title the following Editorial appears in the *N. Y. Med. Record*, and refers to recent meetings of Dental Associations: The medical profession is interested in their deliberations, chiefly on account of the efforts they are making to elevate their standard of education by association in some form or other with medical schools. Each State and local dental society will doubtless discuss the same important question in due time. In the present condition of things, the dentist is puzzled to know whether he is practising a specialty which is simply an offshoot of the great domain of medicine, or whether it is a distinct science and art, independent and standing on its own merits. In other words, a preference is expressed by many of these gentlemen to develop into something more than mere mechanical workers in enamel and dentine. To be oral surgeons savors of much greater dignity, and gives them a decidedly more elevated professional status. Many of the more accomplished among them are sincerely anxious that their duties shall be amplified, so that they shall be something more than handlers of mallets and files, ruthless destroyers of aching nerves, fillers of decayed cavities and extractors of worthless snags of once valuable ivories. Hence has arisen the question for discussion in dental societies everywhere whether dentistry shall not be taught in medical schools. Practical steps have indeed been already taken to test the feasibility of this measure; for we read in the proceedings of the Pennsylvania society, that professors of medical colleges from the State and from New York were personally present, or communicated their views by letter, expressing their desire to add dental chairs to the curriculum of study, so that dental and medical students might be co-educated. It was stated that, so far as the University of Pennsylvania was concerned, nothing but the indifference of its Trustees stood in the way of the accomplishment of this change. It was especially urged by one professor, that the connection of diseases of the teeth and those of the nervous system was well worthy of study in such institutions. We believe that two points must be fully considered previous to a solution of the question. Is it desirable that dentists shall receive a thorough medical education? Is it expedient to teach dental surgery, in all its details, to medical students? So far as we can judge, the dentists seem more anxious about the matter than the doctors. Unless some decided initiatory step is soon taken by a medical school, the fusion may be long postponed, discussion being coupled with inaction from year to year. The terms of the agreement for mutual attachment, by which dental colleges will be absorbed and wholly disappear, will at least be interesting. So much personal sacrifice will be involved, that we confess to some curiosity as to the details by which it is to be effected.

